# Genetic Predictors of Chemotherapy-Induced Peripheral Neuropathy from Paclitaxel, Carboplatin and Oxaliplatin: NCCTG/Alliance N08C1, N08CA and N08CB Study

**DOI:** 10.3390/cancers13051084

**Published:** 2021-03-03

**Authors:** Araba A. Adjei, Camden L. Lopez, Daniel J. Schaid, Jeff A. Sloan, Jennifer G. Le-Rademacher, Charles L. Loprinzi, Aaron D. Norman, Janet E. Olson, Fergus J. Couch, Andreas S. Beutler, Celine M. Vachon, Kathryn J. Ruddy

**Affiliations:** 1 Department of Oncology, Mayo Clinic, Rochester, MN 55905, USA; Adjei.Araba@mayo.edu (A.A.A.); cloprinzi@mayo.edu (C.L.L.); andreas.s.beutler.md@gmail.com (A.S.B.); 2 Alliance Cancer Control Program, Mayo Clinic, Rochester, MN 55905, USA; 3 Department of Quantitative Health Sciences, Mayo Clinic, Rochester, MN 55905, USA; Lopez.Camden@mayo.edu (C.L.L.); schaid@mayo.edu (D.J.S.); jsloan@mayo.edu (J.A.S.); Le-Rademacher.Jennifer@mayo.edu (J.G.L.-R.); olsonj@mayo.edu (J.E.O.); Vachon.Celine@mayo.edu (C.M.V.); Norman.Aaron@mayo.edu (A.D.N.); 4 Alliance Statistics and Data Center, Mayo Clinic, Rochester, MN 55905, USA; 5 Department of Laboratory Medicine and Pathology, Mayo Clinic, Rochester, MN 55905, USA; Couch.Fergus@mayo.edu

**Keywords:** chemotherapy-induced peripheral neuropathy, genome-wide associated study, toxicity

## Abstract

**Simple Summary:**

Chemotherapy-induced peripheral neuropathy (CIPN) is a common debilitating complication of treatment with platinum-based compounds and taxanes. CIPN is predominantly a sensory symptom, causing numbness, tingling, and pain in the hands and/or feet. We performed a genome-wide association study on two independent study groups (N08Cx comprised of NCCTG clinical trial participants in the N08C1, N08CA, and N08CB studies; and Mayo Clinic Breast Disease Registry (MCBDR)) to find genetic variants that are associated with sensory symptoms during or after paclitaxel, paclitaxel and carboplatin, or oxaliplatin receipt. A genetic variant (single nucleotide polymorphism, SNP) rs56360211 near *PDE6C* had a very strong association with CIPN in N08Cx but not in the MCBDR, while the variant rs113807868 near *TMEM150C* was significantly associated with CIPN in the MCBDR but not in N08Cx. This lack of replication suggests that neither is actually strongly associated with CIPN.

**Abstract:**

Chemotherapy-induced peripheral neuropathy (CIPN) is a common and potentially permanent adverse effect of chemotherapeutic agents including taxanes such as paclitaxel and platinum-based compounds such as oxaliplatin and carboplatin. Previous studies have suggested that genetics may impact the risk of CIPN. We conducted genome-wide association studies (GWASs) for CIPN in two independent populations who had completed European Organisation for Research and Treatment of Cancer Quality of Life Questionnaire (EORTC QLQ)-CIPN20 assessments (a CIPN-specific 20-item questionnaire which includes three scales that evaluate sensory, autonomic, and motor symptoms). The study population N08Cx included 692 participants from three clinical trials (North Central Cancer Treatment Group (NCCTG) N08C1, N08CA, and N08CB) who had been treated with paclitaxel, paclitaxel plus carboplatin, or oxaliplatin. The primary endpoint for the GWAS was the change from pre-chemotherapy CIPN20 sensory score to the worse score over the following 18 weeks. Study population The Mayo Clinic Breast Disease Registry (MCBDR) consisted of 381 Mayo Clinic Breast Disease Registry enrollees who had been treated with taxane or platinum-based chemotherapy. The primary endpoint for the GWAS assessed was the earliest CIPN20 sensory score available after the completion of chemotherapy. In multivariate model analyses, chemotherapy regimen (*p* = 3.0 × 10^−8^) and genetic ancestry (*p* = 0.007) were significantly associated with CIPN in the N08Cx population. Only age (*p* = 0.0004) was significantly associated with CIPN in the MCBDR population. The SNP most associated with CIPN was rs56360211 near *PDE6C* (*p* =7.92 × 10^−8^) in N08Cx and rs113807868 near *TMEM150C* in the MCBDR (*p* = 1.27 × 10^−8^). Due to a lack of replication, we cannot conclude that we identified any genetic predictors of CIPN.

## 1. Introduction

Chemotherapy-induced peripheral neuropathy (CIPN) is a common debilitating clinical complication that arises from some common anticancer agents including taxanes, platinum compounds, and vinca alkaloids. CIPN ranks among the most common non-hematological dose-limiting toxicities of the platinum and taxane compounds. CIPN is often a sensory-predominant symptom associated with numbness, tingling, and neuropathic pain, especially in the hands and feet [1,2,3,4]. CIPN can occur acutely during chemotherapy and may require a reduction in drug dosage or even the premature stoppage of a planned treatment course, potentially impairing the efficacy of oncological treatment and survival [5,6]. Acute CIPN often resolves after the completion of chemotherapy, but it can sometimes persist for years, impairing quality of life over the long-term [2,5]. The National Cancer Institute Common Terminology Criteria for Adverse Events (NCI-CTCAE) facilitate the standardized clinician reporting of adverse events [2,7], but patient-reported outcomes have been demonstrated to be more accurate and sensitive than CTCAE measures for understanding cancer treatment outcomes in general and true CIPN burden specifically [8,9,10,11,12,13,14,15]. The European Organisation for Research and Treatment of Cancer Quality of Life Questionnaire (EORTC QLQ) CIPN20 is a questionnaire used to assess patient-reported severity of CIPN [2,16,17]. The prevalence of CIPN varies between different chemotherapy agents including platinum compounds and taxanes (two of the most commonly used classes of chemotherapy drugs); incidence rates range from 19 to 85% [7]. Reports have indicated that after the cessation of chemotherapy, CIPN is observed in nearly 70% of patients in the first month, in 60% at three months, and in 30% of patients at six months; as such, the concern that CIPN may cause long-lasting debility is a common reason for chemotherapy cessation or drug dose reduction [1,2,5,7,18]. Wide variations in CIPN severity between individuals receiving identical chemotherapy regimens suggest that genetic predisposition may play a role [1].

Pharmacogenomics research to identify genetic variation associated with CIPN, including markers specific to paclitaxel-induced peripheral neuropathy (PIPN) or oxaliplatin-induced peripheral neuropathy (OIPN), have been reported through candidate gene and genome-wide association studies (GWASs) [1,2,18,19,20,21,22,23,24,25,26,27,28,29,30,31,32,33]. Polymorphisms including rs7349683 in *EPHA5* [21,26], rs10509681 and rs11572080 in *CYP2C8* [34], and rs3213619 in *ABCB1* [19] have been reported to be associated with the risk of PIPN. Other studies have not been able to validate some of these associations for SNPs (single nucleotide polymorphism) in *ABCG2, ACYP2, BTG4, CCNH, FARS2*, and *FOXC1* with OIPN [25,30,33,35].

The rs9657362 and rs17683288 genetic variants in a Charcot–Marie–Tooth disease (CMT) gene, *ARHGEF10*, were found to be associated with protection against PIPN, while a risk effect was associated with SNP rs2294039. These SNPs were found through the targeted DNA sequencing of patients with an extreme phenotypes for PIPN (extremely severe versus extremely little PIPN) in two North Central Cancer Treatment Group (NCCTG) trials, N08C1 and N08CA [36,37]. A recent study with oxaliplatin-treated patients, N08CB, failed to associate any CMT-related genetic polymorphisms with OIPN susceptibility [38].

The present study assessed patient-reported CIPN data from patients who had received a taxane (paclitaxel), platinum compound (oxaliplatin), or both drug classes (paclitaxel and carboplatin), and it performed two separate GWAS analyses—one on a clinical trial population and the other on a clinical cohort population. The aim of the study was to identify genetic variants that influence CIPN from paclitaxel and platinum-based therapy.

## 2. Materials and Methods

### 2.1. Patient Population

The clinical trial population, herein referred to as N08Cx, comprised participants in the N08C1 (paclitaxel and carboplatin treatment), N08CA (paclitaxel treatment), and N08CB (oxaliplatin treatment) NCCTG clinical trials for whom serial patient-reported neuropathy assessments had been obtained using the CIPN20 [39,40,41]. NCCTG is now part of the Alliance for Clinical Trials in Oncology. The NCCTG N08C1 study, as described previously [36,41,42], was designed to study the natural history of paclitaxel neuropathy and to test some genetic correlatives in patients with various cancers including breast, ovarian, and lung. The 284 patients on study were treated with paclitaxel at a dose of 70–90 mg/m^2^ weekly or 175 mg/m^2^ every 3 weeks, with or without carboplatin. The N08CA study, as previously described [37,39,43], included 185 patients who received carboplatin (CBDCA) at area under the curve (AUC) = 5–7 every 21 or 28 days for at least 12 weeks with paclitaxel given either 150–200 mg/m^2^ every 21–28 days or 80 mg/m^2^ weekly for at least 12 weeks. Patients were also randomly assigned to receive 1.5 g/m^2^ of glutathione or placebo (100 mL of 0.9% NaCl) intravenously prior to chemotherapy for the possible prevention of paclitaxel/carboplatin-induced peripheral neuropathy. The third clinical trial, N08CB, studied intravenous calcium (Ca) and magnesium (Mg) for the prevention of oxaliplatin-induced neurotoxicity in colon cancer patients [38,40,43,44]. In this randomized trial, 353 patients with colon cancer were randomly assigned to one of three arms: (1) Ca/Mg before and after oxaliplatin-containing chemotherapy, (2) Ca/Mg before and placebo after oxaliplatin-containing chemotherapy, or (3) placebo before and after oxaliplatin-containing chemotherapy [40]. In all three trials, patients were at least 18 years old and were excluded if they had a pre-existing history of peripheral neuropathy (except Grade 1 baseline CIPN was allowed in N08CA), other medical conditions that could have interfered with study participation, prior treatment with paclitaxel and/or carboplatin, or the concurrent use of agents for neuropathy prevention.

**Figure 1:**
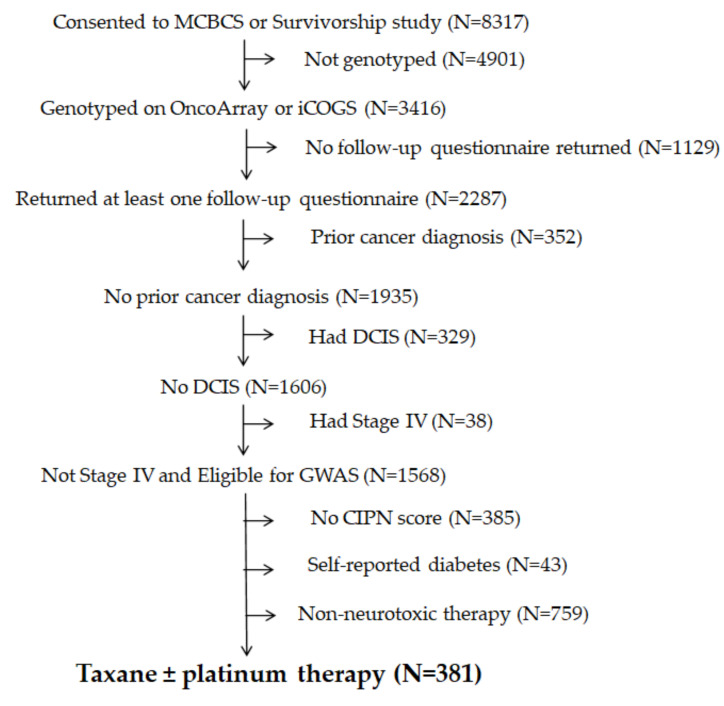
Breast registry flow diagram. DCIS: ductal carcinoma in situ; GWAS: genome-wide association study; CIPN: chemotherapy-induced peripheral neuropathy; MCBCS: Mayo Clinic Breast Cancer survey; iCOGS: Illumina iSelect genotyping array, designed as part of the Collaborative Oncological Gene-Environment Study (COGS).

A separate clinical observational cohort was made up of breast cancer patients from the Mayo Clinic Breast Disease Registry (MCBDR), an ongoing longitudinal cohort that enrolls patients diagnosed with breast cancer within the prior year. Participants completed questionnaires at baseline and during follow-up, approximately annually (by mail), and allowed for reviews of their medical records and access to tumor tissue when available. More than 8000 patients have consented to participate in the MCBDR since 2003, with accrual rates currently approximating 600/year (70–80% of those approached). Patients with prior cancers, ductal carcinoma in situ (DCIS)/stage 0, stage-4/metastatic breast cancer, and self-reported diabetes were excluded from the GWAS analysis, and the patients included in the analyses were those who returned at least one follow-up questionnaire that included EORTC QLQ-CIPN20, who had received a paclitaxel and platinum compound, and for whom genotyping had been performed (Figure 1). All the participants in these studies signed an Institutional Review Board (IRB)-approved informed consent, in accordance with federal and institutional guidelines.

### 2.2. Measurement of Chemotherapy-Induced Peripheral Neuropathy (CIPN) Symptoms

CIPN was measured on all studies using the QLQ-CIPN20 questionnaire [39,40,41,42]. This 20-item questionnaire includes three scales that evaluate sensory, autonomic, and motor symptoms. Patients rate their experience for each aspect of CIPN using scores from 1 (not at all) to 4 (very much). The questionnaire has been tested in cancer patients receiving a variety of chemotherapy agents and has been shown to have internally consistent reliability [42,43,45]. While the autonomic and motor subscales have been found to suboptimally correlate with changes in CIPN symptoms, the sensory subscale is highly correlated [16,43]. For N08Cx, the QLQ-CIPN20 was completed by patients prior to each dose of chemotherapy. The primary outcome of the N08Cx GWAS was chosen as the change in the CIPN20 sensory score from baseline to the worst score (representing the most severe symptoms) reported within 18 weeks of baseline, with only those patients who had at least two post-baseline score measurements included. For the MCBDR, no CIPN20 data were collected before chemotherapy because the CIPN20 instrument was not included in the baseline survey and also because patients may enroll in that cohort study up to a year after diagnosis. Because pre-chemotherapy CIPN20 scores were not routinely collected, it was not possible to assess change in scores to mimic the N08Cx primary outcome. The CIPN20 instrument was also not part of every follow-up questionnaire, and some participants did not respond to every questionnaire. Therefore, the primary endpoint for the MCBDR GWAS was not based on any score change over time, but rather on the earliest available CIPN20 sensory score during follow-up after receipt of chemotherapy. We re-scaled the CIPN20 scores in both populations so that 0 represented the most severe symptoms and 100 represented no symptoms, as specified in the analysis plan of the N08CX trials protocols [39,40,46] and similar to scaling used previously [46]. Hence, a negative change from baseline in N08Cx corresponded to worsening of symptoms, and a lower score corresponded to worse symptoms in both N08Cx and the MCBDR.

### 2.3. DNA Extraction, Genotyping and Quality Control

Genomic DNA was extracted from blood samples collected as part of the clinical trial (N08Cx) and as part of the registry (MCBDR). Genotyping for the N08Cx study samples was performed on Illumina’s Infinium Human OmniExpress (https://www.illumina.com) at the Mayo Clinic Medical Genome Facility. The MCBDR samples were genotyped on either of two platforms, the Illumina Infinium Onco Array (https://www.illumina.com) or the Illumina iSelect genotyping array (iCOGS) chip—a platform specifically designed to evaluate genetic variants associated with the risk of breast, ovarian, and prostate cancer (http://www.cogseu.org/) [47,48,49]. All of the genotype data were received 1 April 2020.

For quality control purposes, the genotype data were cleaned to remove unmapped SNPs, duplicate samples, samples with inconsistency between reported sex and genetic data or closely related kinship (within first degree relatives) according to KING [50], and SNPs with call rates <98%. SNPs with a minor allele frequency <5% were removed because of limited statistical power for such SNPs. The STRUCTURE software [51] was used to determine the genetic ancestry admixture for the patients of the study and reference samples (n = 585) of known ancestry from the 1000 Genome database that served as population anchors. A single primary ancestry category (African, Asian, or Caucasian) was predicted for each study sample. Principal component analysis was utilized to assess and correct for population stratification. To increase the genome coverage, genotypes (allele dosages) were imputed by the University of Michigan imputation server [52]. SNPs with an imputation r^2^ < 0.3 were excluded. For the MCBDR GWAS, only SNPs common to the OncoArray- and iCOGS-derived datasets (after other data processing steps) were considered.

### 2.4. Candidate Gene SNPs

Several reviews on studies testing associations between SNPs from candidate genes and neuropathy have been published [19,21,22,26,28,33,36,37,51,52,53,54]. In these studies, CIPN severity has been graded mostly by the NCI-CTCAE, by Functional Assessment of cancer therapy (FACT)-Taxane or Total Neuropathy Score (TNS), or by EORTC-CIPN20 scores [2,20,55]. To validate previously reported associations, we examined those SNPs (from previous candidate gene studies) with changes in baseline CIPN20 scores (N08Cx) and with the absolute earliest CIPN score at follow-up (MCBDR).

### 2.5. Statistical Analysis

Linear regression was used to determine potential covariates to be adjusted for in analyses, as well as to test the association between each SNP and the trait of interest, adjusted for selected covariates (*p* < 0.1). SNP genotypes were represented by the dose of the minor (alternate) allele with an additive model for the allele effect, and genome-wide significance was defined as *p* < 5 × 10^−8^ [56,57]. Quantile–quantile (Q–Q) plots were used to visually evaluate whether population stratification was controlled by plotting the distribution of observed *p*-values versus the distribution expected under a null hypothesis of no SNP associations. Manhattan plots were used to plot *p*-values for all SNP associations across chromosomes, and regional association plots (LocusZoom) [58] were used to provide detail on genetic regions of interest, providing gene annotations and pairwise correlations between the surrounding SNPs and the SNP of interest.

## 3. Results

### 3.1. Patient Characteristics

Table 1 shows the demographics of 692 patients who were enrolled in the GWAS for N08Cx with at least two post-baseline CIPN20 scores and genetic samples that passed quality control. There were 71.1% women and 28.9% men; the mean age was 57.6 years. Most patients (85.7%) self-reported their race as White, while 10.5% self-reported as Black or African American, and 2.6% self-reported as Asian. The self-reported race data generally agreed with the genetic-based ancestry category with a few exceptions mainly amongst those who reported themselves to be non-White. Nine patients (including five American Indian/Alaska Native, two African American, and two Asian by self-reporting) were categorized as having primarily Caucasian genetic ancestry. Only 2.5% of N08Cx patients reported a Grade 1 baseline neuropathy (asymptomatic; loss of deep tendon reflexes or paresthesia, NCI CTCAE v4). The mean CIPN20 sensory score in N08Cx at baseline was 97.1 (on a scale of 0–100, where lower scores corresponded to worse symptoms and higher scores corresponded to less severe symptoms). Diabetes status was only reported in the N08CA study, while body mass index (BMI) was only reported in the N08CA and N08CB studies. The primary outcome of the N08Cx study was a change from baseline to worst post-baseline score within 18 weeks of baseline. We considered the following as potential covariates: patient sex, age, baseline Eastern Cooperative Oncology Group (ECOG) performance status, chemotherapy regimen, genetic ancestry category, and the first five principal components of the genetic data.

The factors most strongly associated with the primary outcome and selected as covariates were sex, chemotherapy regimen, and genetic ancestry. In the multivariate analysis (Table 2), oxaliplatin treatment was used as the comparator and the severity of CIPN (coefficient estimate (CE), standard error (SE), and *p*-value (*p*)) was observed to be worse in the biweekly paclitaxel treatment arm (CE = −15.32, SE = 3.93, and *p* = 0.0001) than in the weekly paclitaxel alone (CE = −9.01, SE = 2.55, and *p* = 0.0004) or every three weeks paclitaxel/carboplatin combination (CE = −8.26, SE = 1.82, and *p* = 6.77 × 10^−6^). Overall, the chemotherapy regimen was significantly associated with CIPN (*p* = 3.0 × 10^−8^).

Furthermore, genetic ancestry, specifically African American ancestry, was also significantly associated with CIPN change from baseline (CE = −7.63, SE = 2.43, and *p* < 0.002) with an overall significance of *p* = 0.007, while sex was only borderline significant (CE = 3.29, SE = 1.77, and *p* = 0.06), Table 2.

At the time of this study, 8317 patients had consented to participate in the MCBDR (Figure 1). After the exclusion of subjects without genotyping data (n = 4901), those not returning at least one follow-up questionnaire that included CIPN20 (n = 1129), those who had been diagnosed with cancer previously (and therefore might have received additional treatments, n = 352), those with DCIS/stage 0 (n = 329) or stage 4/metastatic disease (n = 38 as previously described in Section 2.1), those who reported having diabetes (n = 43), and those who did not return a questionnaire with a complete CIPN20 (n = 385), a total of 1140 patients remained (Figure 1).

Out of this number, 381 had undergone paclitaxel, paclitaxel and carboplatin, or oxaliplatin treatment and had CIPN20 data scores (Figure 1 and Table 3) and were included in the GWAS. The CIPN20 was collected on two follow-up questionnaires, one sent approximately three years after initial cancer diagnosis and another sent between 1 and 17 years after initial cancer diagnosis (depending on when the patient enrolled in the MCBDR). Patients included in our GWAS completed the CIPN20 a median of 7.1 years after diagnosis (range: 1.8–15.7). As potential covariates, we considered the following: patient age, years since cancer diagnosis (as a proxy for years since treatment), treatment with a platinum agent, genotyping platform (OncoArray or iCOGS), and the first five genetic principal components. Patient sex and ancestry category were not considered because only one patient was male, and all were categorized as Caucasian.

We used all of the candidate covariates in the analysis except for the non-significant principal components in the GWAS. Only older age was statistically significantly associated with worse CIPN20 scores in the multivariate model (*p* = 0.0004); see Table 4.

### 3.2. Genome-Wide Association Study (GWAS) Results

**Figure 2:**
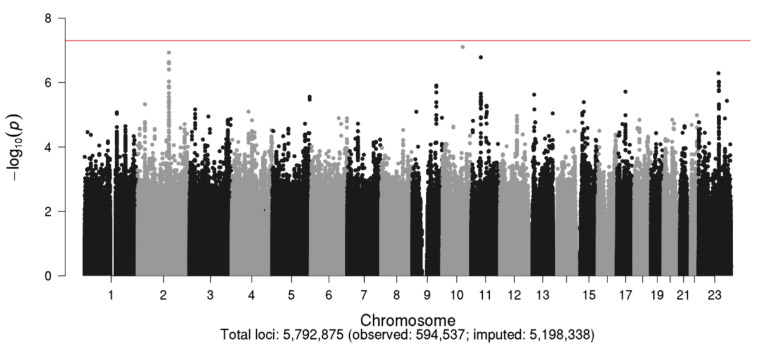
Manhattan plot for the N08Cx study.

**Figure 3:**
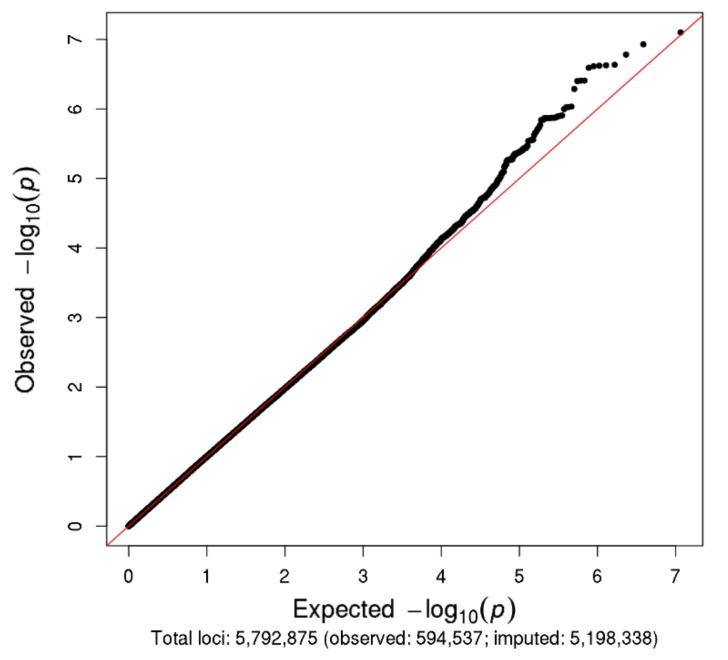
Q–Q plot for N08Cx study.

The Manhattan and Q–Q plots for the N08Cx GWAS study are shown in Figure 2 and Figure 3, respectively. Though no SNPs achieved genome-wide significance (*p* < 5.0 × 10^−8^), the rs5636021 SNP on chromosome 10 near the *PDE6C* gene approached this level of significance (*p* = 7.92 × 10^−8^).

The SNPs with *p* < 1.0 × 10^−6^ are shown in Table 5. One SNP on chromosome 11 (rs10769096), two SNPs on the X chromosome (rs4969675 and rs73538805), and 11 SNPs located within the same intergenic 2q22 chromosome region (a gene desert) had *p*-values < 1.0 × 10^−6^. Using LDlink [59,60], a suite of web-based applications designed to easily and efficiently interrogate linkage disequilibrium in population groups, all of the chromosome 2 SNPs were determined to be in strong linkage disequilibrium (LD) (r^2^ = 0.913–1.0 and D’ = 0.977–1.0), as observed from the Caucasian population. D’ (D prime) values range from 0 to 1 with higher values indicating tight linkage of alleles.

**Figure 4:**
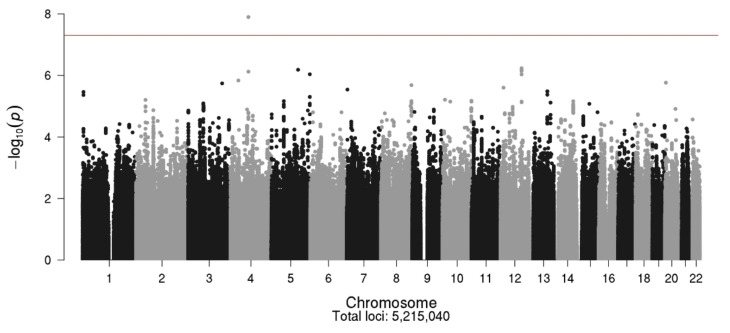
Manhattan plot for the MCBDR study.

**Figure 5:**
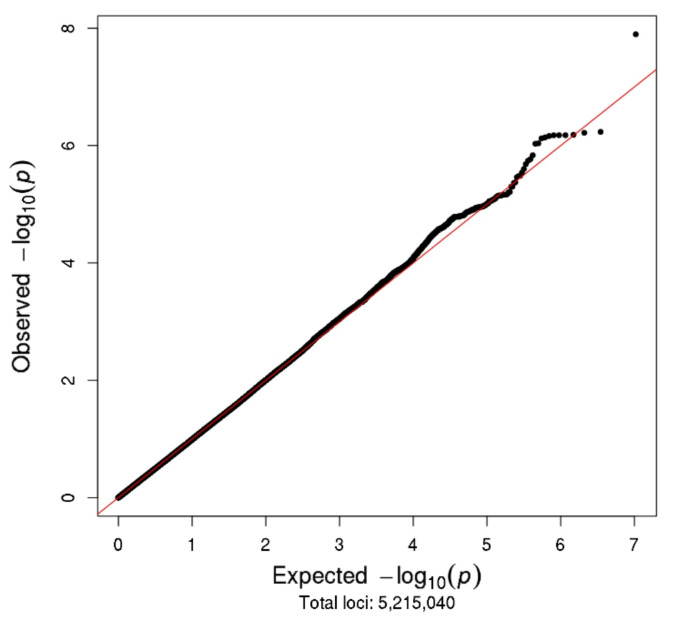
Q–Q plot for the MCBDR study.

The Manhattan plot for the MCBDR GWAS study is shown in Figure 4, with the corresponding Q–Q plot shown in Figure 5. The strongest association with CIPN was with SNP rs113807868 (*p* = 1.27 × 10^−8^) on chromosome 4 near the *TMEM150C* gene.

Another SNP near this gene (rs2868379) showed evidence of association (*p* = 7.54 × 10^−7^). Additional SNPs with *p* < 1.0 × 10^−6^ are shown in Table 6. Two SNPs on chromosome 5 and eight other SNPs on chromosome 12 were also associated with CIPN at a marginal significance level of *p* < 1.0 × 10^−6^. In the Caucasian population, all the chromosome 12 SNPs (Table 6) were found to be in strong LD with one another (r^2^ and D’ = 1) [59,60]. SNP positions on chromosomes were based on the GRCh37 (Genome Reference Consortium Human Build 37) assembly in National Center for Biotechnology Information (NCBI).

### 3.3. Candidate SNP Analysis

Based on prior studies, we performed focused analyses of candidate SNPs that have been reported to be associated with CIPN among patients treated with either paclitaxel or platinum-based drugs. The results in Table 7 and Table 8 showed that none of the candidate SNPs were statistically significantly associated with CIPN in either of the two cohorts (with a Bonferroni adjusted significance threshold of approximately = 0.001). Out of the 74 statistical tests presented in Table 7 and Table 8, four had *p*-values < 0.05, as would be expected by random chance.

## 4. Discussion

Chemotherapy-induced peripheral neuropathy can lead to early treatment discontinuation, potentially reducing treatment efficacy and the quality of life. To date, several genetic studies have linked SNP markers to risk of CIPN. Independent studies are critical to replicate these findings before they are used to inform cancer treatment decisions. Several studies have identified genetic polymorphisms associated with neuropathy during the receipt of taxane or platinum-based chemotherapies, but larger replication studies have often failed to confirm these associations. In this study, we performed a GWAS to identify genetic variants that are associated with CIPN in two independent study populations—one was a combined group of patients from three clinical trials (N08Cx) who were treated with paclitaxel and carboplatin (N08C1), paclitaxel and carboplatin (N08CA), or oxaliplatin (N08CB), and the second was a cohort of patients participating in a longitudinal MCBDR.

In the N08Cx study, we observed that chemotherapy regimen, particularly the combination therapy of paclitaxel and carboplatin and how often the chemotherapy was given (treatment scheduled), were significantly associated with worse CIPN, as was African ancestry. In the MCBDR cohort, older age was associated with worse CIPN. These results confirmed previous findings from other studies [66,67,68]. One SNP, rs113807868, was associated with CIPN at genome-wide significance only in the MCBDR population. No common SNP was identified between N08Cx and the MCBDR for association with CIPN. The SNP with strongest association with CIPN in the N08Cx study was rs56360211 (*p* = 7.92 × 10^−8^), an intron variant in *PDE6C*, a phosphodiesterase 6C gene that encodes a subunit of cone phosphodiesterase. This SNP is located on chromosome 10, and mutations in *PDE6C* have been linked to cone dystrophy type 4 (CODA) and achromatopsia, conditions that result from a loss of cone function characterized by low visual acuity, a lack of color discrimination, and excessive sensitivity to light with a sensation of discomfort or pain in the eyes [69]. The other SNP that was linked to CIPN in the N08Cx study is rs10769096, located in *TSPAN18* (tetraspanin 18 gene), a gene that encodes a member of membrane proteins with four transmembrane (tetraspanin) domains that are involved in cellular penetration, adhesion, motility, and signal conduction [70,71,72]. Polymorphisms in *TSPAN18* have been associated with schizophrenia, but these associations have not been consistently replicated [73,74,75,76]. The SNP identified to be associated with CIPN with genome-wide significance in the MCBDR population, rs113807868, is located in *TMEM150C* on chromosome 4, a gene that encodes a transmembrane protein component of a mechanosensitive ion channel that is activated by mechanical stimuli in various cell types and confers slowly adapting, mechanically activated currents in dorsal root ganglion neurons. Mechanically activated ion channels are sensors that are critical for hearing, touch, pain, and blood pressure regulation, and the absence of this gene in mice has been found to be associated with muscle weakness and a loss of motor coordination [77]. Though *TMEM150C* has not been previously identified to be associated with CIPN, it is biologically plausible that its impact on ion channels and pain could mediate neuropathy. Taxane treatment has been reported to affect the dorsal root ganglion and neuron cell bodies of peripheral nerves [78,79].

We did not find any of the previously reported candidate SNPs to be strongly linked to the sensory subdomain of CIPN from paclitaxel or platinum [19,21,22,26,33,36,37,53,61,62,63,64,65] in either the N08Cx or MCBDR GWAS analyses.

We also explored the heritability of the traits (results not shown) by using all SNPs and found the estimated heritability for both the CIPN20 sensory change from baseline in the N08Cx study and the CIPN20 sensory at follow-up in the MCBDR study to be 0%. This suggested that there is little evidence that either of these traits has strong genetic etiology. However, there are limitations in these estimated heritabilities. First, our sample sizes did not provide precise estimates of heritability, with a 95% upper confidence limit of 53% for the heritability in the N08Cx study and a nearly 100% heritability upper limit for the MCBDR study. Second, heritability based on GWAS assumes there are a large number of causal variants, each of small effect. If in fact there were just one or a few causal variants, then the estimated heritability would likely be near 0% because the majority of SNPs used in the calculations would drown out the signal from a few variants.

The limitations of this study included the heterogeneity of time points for the assessment of CIPN and the treatments received. Though the CIPN20 sensory tool was used to measure CIPN for both studies, the N08Cx populations had CIPN scores derived from worse change in baseline within 18 weeks of treatment and the MCBDR population had the CIPN score measured long after treatment in many cases (and received treatments were very variable, as occurs outside of clinical trials). This was because CIPN20 was not collected before, during, or shortly after receipt of chemotherapy in the MCBDR for most participants. The MCBDR is a longitudinal cohort study that intermittently surveys patients over time (generally no more often than annually and sometimes not before treatment starts because patients can be enrolled up to a year after their breast cancer diagnosis). This contrasts with the N08Cx assessments, which occurred before and during chemotherapy because they were critical to the primary endpoint of these three trials. In addition, the autonomic and the motor subscales of the EORTC-CIPN20 were not used in this study, but they may have produced different results, as might other measures of CIPN, including single-item measures that have been demonstrated to be more global and sensitive to change and related to numerous genetic variables with replication [12,80,81]. In addition, we were limited by the small sample sizes (which limit statistical power to detect small or moderate genetic effects) of the available N08Cx and MCBDR GWAS populations; this is a common issue in pharmacogenomics studies [76,82].

## 5. Conclusions

In summary, we performed separate CIPN GWAS studies in two independent patient populations treated with paclitaxel and carboplatin or oxaliplatin chemotherapy, and we identified one genome-wide significant SNP in one of the two populations. This SNP has not been previously reported to be associated with CIPN. Because of a lack of replication between the two populations (N08Cx and MCBDR) and a failure to achieve genome-wide significance (i.e., *p* < 5.0 × 10^−8^) for all but one SNP, we conclude that we have not identified any SNPs that are definitively associated with CIPN.

## Figures and Tables

**Table 1:** Baseline characteristics of the N08Cx study cohort.

Characteristic		Study (Participants ^a^)			
		N08C1 (246)	N08CA (150)	N08CB (296)	Total (692)
Age	Mean (SD)	56.5 (11.4)	61.0 (10.4)	56.7 (11.3)	57.6 (11.3)
	Median (Q1, Q3)	56.0 (48.2, 64.0)	61.5 (55.0, 68.0)	56.0 (50.0, 65.0)	57.0 (50.0, 65.2)
	Range	23.0–85.0	28.0–85.0	24.0–83.0	23.0–85.0
Sex (%)	Female	213 (86.6%)	122 (81.3%)	157 (53.0%)	492 (71.1%)
	Male	33 (13.4%)	28 (18.7%)	139 (47.0%)	200 (28.9%)
Race (%)	White	202 (82.1%)	139 (92.7%)	252 (85.1%)	593 (85.7%)
	Black/African American	30 (12.2%)	9 (6.0%)	34 (11.5%)	73 (10.5%)
	Native Hawaiian or Other Pacific Islander	0 (0.0%)	0 (0.0%)	0 (0.0%)	0 (0.0%)
	Asian	11 (4.5%)	2 (1.3%)	5 (1.7%)	18 (2.6%)
	American Indian or Alaska Native	1 (0.4%)	0 (0.0%)	3 (1.0%)	4 (0.6%)
	Not Reported	2 (0.8%)	0 (0.0%)	1 (0.3%)	3 (0.4%)
	Unknown	0 (0.0%)	0 (0.0%)	1 (0.3%)	1 (0.1%)
Body Mass Index	Missing data ^b^	246	0	1	247
	Mean (SD)	NA	27.7 (8.0)	28.9 (7.1)	28.5 (7.4)
	Median (Q1, Q3)	NA	25.5 (22.2, 30.5)	27.6 (24.4, 31.9)	26.9 (23.6, 31.8)
	Range	NA	16.7–62.5	15.3–64.8	15.3–64.8
ECOG Performance Status (PS), (%)	0	152 (61.8%)	70 (46.7%)	195 (65.9%)	417 (60.3%)
	1	94 (38.2%)	70 (46.7%)	98 (33.1%)	262 (37.9%)
	2	0 (0.0%)	10 (6.7%)	3 (1.0%)	13 (1.9%)
Baseline Neuropathy- National Cancer Institute Common Terminology Criteria for Adverse Events (NCI CTCAE) v4 (%)	None	246 (100.0%)	133 (88.7%)	296 (100.0%)	675 (97.5%)
	Grade 1	0 (0.0%)	17 (11.3%)	0 (0.0%)	17 (2.5%)
Baseline CIPN20 Sensory Score	Mean (SD)	97.3 (5.1)	94.7 (8.7)	98.1 (5.0)	97.1 (6.2)
	Median (Q1, Q3)	100.0 (96.3, 100.0)	100.0 (92.6, 100.0)	100.0 (100.0, 100.0)	100.0 (96.3, 100.0)
	Range	70.4–100.0	33.3–100.0	44.4–100.0	33.3–100.0
Cancer Type (%)	Breast	143 (58.1%)	ND	0	ND
	Lung	38 (15.4%)	43 (28.7%)	0	81 (11.7%)
	^c^ Ovarian	36 (14.6%)	54 (36.0%)	0	90 (13.0%)
	^d^ Colorectal	ND	ND	296 (100.0%)	ND
	Other	29 (11.8%)	39 (26.0%)	0	ND
	Not reported	0	14 (9.3%)	0	14 (2.0%)

^a^ Demographics for 692 patients on study with GWAS and CIPN20 sensory data; ^b^ body Mass is not reported for N08C1 study; ^c^ ovarian: includes peritoneal and fallopian cancers; ^d^ colorectal: includes adenocarcinoma of the colon or rectum; NA: not applicable; ND: not determined; ECOG: Eastern Cooperative Oncology Group; CIPN20: CIPN-specific 20-item questionnaire.

**Table 2:** Multivariate linear regression analysis.

Clinical Feature	Covariate	Coefficient		*p*-Value	Overall *p*-Value
		Estimate	SE		
Sex	Female (reference)	-	-	-	-
	Male	3.29	1.77	0.06	
Chemotherapy Regimen	Oxaliplatin 2 wk (reference)	-	-	-	3.0 × 10^−8^
	Pac Only 1 wk	−9.01	2.55	0.0004	
	Pac Only 2 wk	−15.32	3.94	0.0001	
	Pac Only 3 wk	−7.23	6.02	0.23	
	Pac and Carb 1 wk	3.71	2.66	0.16	
	Pac and Carb 3 wk	−8.26	1.82	6.77 × 10^−6^	
Genetic Ancestry	European (reference)	-	-	-	0.007
	African	−7.63	2.43	0.002	
	Asian	0.98	4.86	0.84	

Factors associated with CIPN20 (N08Cx study population) measured as change from baseline to minimum CIPN20 sensory score (corresponding to most severe symptoms) within 18 weeks of baseline, Overall *p*-value derived from Wald significance test, Pac: paclitaxel; Carb: carboplatin; SE: standard error.

**Table 3:** Baseline characteristics of the Mayo Clinic Breast Disease Registry (MCBDR) GWAS cohort.

Characteristics of Participants (n = 381)			
		Cancer Diagnosis	CIPN Reported
Age	Mean (SD)	48.4 (9.5)	55.5 (10.8)
	Median (SD)	48.4 (42.2–53.8)	56.0 (48.8–62.4)
	Range	24.9–82.7	28.6–87.6
Sex (Gender)	Female	380 (99.7%)	
	Male	1 (0.3%)	
Race	White	374 (98.2%)	
	Non-white, unknown, or undisclosed	7 (1.8%)	
Body Mass Index (N)	Missing data	32	
	Mean (SD)	27.9 (5.7)	
	Median (Q1, Q3)	27.0 (23.9, 31.1)	
	Range	15.8–46.0	
Years from Cancer Diagnosis to CIPN20	Mean (SD)	7.1 (3.7)	
	Median (Q1, Q3)	7.2 (3.2, 10.0)	
	Range	1.8–15.7	
Treatment	Taxane only	342 (89.8%)	
	Taxane and platin	39 (10.2%)	
Genotyping Platform	OncoArray	233 (61.2%)	
	iCOGS	148 (38.8%)	

**Table 4:** Multivariate linear regression.

Clinical Feature	Covariate	Coefficient		*p*-Value
		Estimate	SE	
Age (Years)		−0.31	0.09	0.0004
Years from Cancer Diagnosis to CIPN20		0.33	0.35	0.34
Treatment	Taxane only	-	-	-
	Taxane and platin	−1.28	2.7	0.64
Genotyping Platform	OncoArray	-	-	-
	iCOGS	−1.15	2.24	0.64

Factors associated with CIPN20 (MCBDR cohort population). SE: standard error.

**Table 5:** SNPs with *p*-value < 1 × 10^−6^ associated with change in CIPN20 sensory in the N08Cx study population.

RSID	^1^ Gene	Chr	Position (bp)	Allele		Sample numbers (Genotype)			Minor Allele Freq (alt.freq)	Coefficient		*p*-value	*p*-value in MCBDR
				Ref -Major	Alt -Minor	Major -ref/ref	Hetero-zygous -ref/alt	Variant -alt/alt		Estimate	SE		
rs56360211	*PDE6C*	10	95374453	T	G	623	66	3	0.054	−12.92	2.38	7.92 × 10^−8^	0.34
rs1515252	-	2	146779243	G	A	314	302	76	0.328	−5.96	1.11	1.18 × 10^−7^	0.07
rs10769096	*TSPAN18*	11	44943260	G	A	479	180	33	0.174	−7.74	1.46	1.65 × 10^−7^	0.82
rs16825861	-	2	146742030	G	A	318	301	73	0.323	−5.87	1.12	2.32 × 10^−7^	0.11
rs13026986	-	2	146753902	T	G	318	301	73	0.321	−5.91	1.13	2.36 × 10^−7^	0.11
rs1606806	-	2	146758685	C	T	317	302	73	0.322	−5.91	1.13	2.39 × 10^−7^	0.10
rs34053477	-	2	146742600	G	A	318	301	73	0.321	−5.90	1.13	2.43 × 10^−7^	0.11
rs16825916	-	2	146768842	A	G	317	301	74	0.322	−5.88	1.13	2.56 × 10^−7^	0.10
rs7583107	-	2	146819721	C	T	71	325	296	0.663	5.74	1.12	3.91 × 10^−7^	0.04
rs4662450	-	2	146806610	A	C	71	325	296	0.659	5.77	1.13	3.92 × 10^−7^	0.05
rs7565993	-	2	146803053	A	G	71	325	296	0.659	5.77	1.13	3.97 × 10^−7^	0.05
rs4969675	-	X	95803439	T	C	15	244	433	0.788	13.27	2.62	5.16 × 10^−7^	-
rs36049952	-	2	146839632	T	C	346	286	60	0.292	−5.84	1.18	9.25 × 10^−7^	0.04
rs12991309	-	2	146822623	G	A	346	286	60	0.292	−5.83	1.18	9.38 × 10^−7^	0.04
rs73538805	-	X	97724436	T	C	486	186	20	0.162	7.02	1.42	9.42 × 10^−7^	-

^1^*PDE6C:* phosphodiesterase 6C gene; *TSPAN18:* tetraspanin 18 gene. All chromosome 2 SNPS are in linkage disequilibrium; Chr: chromosome, SE: standard error; Freq: frequency. SNP positions on chromosome were based on the GRCh37 (Genome Reference Consortium Human Build 37) assembly in National Center for Biotechnology Information (NCBI); RSID: Reference SNP cluster ID; SNP: single nucleotide polymorphism.

**Table 6:** SNPs with *p*-value < 1 × 10^−6^ associated with CIPN in the MCBDR cohort population.

RSID	^1^ Gene	Chr	Position (bp)	Allele		Sample Numbers (Genotype)			Minor Allele Freq (alt.freq)	Coefficient		*p*-Value	*p*-Value in N08Cx
				Ref -Major	Alt -Minor	Major -ref/ref	Hetero-zygous -ref/alt	Variant -alt/alt		Estimate	SE		
rs113807868	*TMEM150C*	4	83439324	G	A	346	32	3	0.068	−16.18	2.78	1.27 × 10^−8^	0.77
rs78825864	-	12	98169501	C	A	316	60	5	0.093	−9.76	1.92	5.84 × 10^−7^	0.18
rs77885228	-	12	98174620	A	G	315	61	5	0.095	−9.67	1.91	6.05 × 10^−7^	0.10
rs77880756	-	5	124192458	G	A	280	96	5	0.143	−9.08	1.79	6.54 × 10^−7^	0.54
rs76151599	-	12	98169090	T	C	315	61	5	0.095	−9.64	1.91	6.65 × 10^−7^	0.10
rs76505485	-	12	98169019	A	C	315	61	5	0.095	−9.64	1.91	6.65 × 10^−7^	0.10
rs76175313	-	12	98167034	C	T	315	61	5	0.095	−9.64	1.91	6.67 × 10^−7^	0.11
s75111732	-	12	98161392	T	C	315	61	5	0.095	−9.65	1.91	6.88 × 10^−7^	0.11
rs11109196	-	12	98155827	A	G	315	61	5	0.095	−9.65	1.91	7.25 × 10^−7^	0.09
rs2868379	*TMEM150C*	4	83438645	T	C	325	52	4	0.104	−11.35	2.26	7.54 × 10^−7^	0.61
rs4331859	LOC105377763	5	179094108	A	C	266	106	9	0.163	−7.55	1.51	9.17 × 10^−7^	0.04
rs12317534	LOC643711	12	98139760	A	C	312	64	5	0.098	−9.55	1.91	9.31 × 10^−7^	0.07

^1^*TMEM150C*: transmembrane protein 150C. All the chromosome 12 SNPS are in linkage disequilibrium. Chr: chromosome; SE: standard error; Freq.: frequency. SNP positions on chromosome were based on the GRCh37 (Genome Reference Consortium Human Build 37) assembly in National Center for Biotechnology Information (NCBI), RSID: Reference SNP cluster ID.

**Table 7:** Candidate SNPs associated with CIPN from paclitaxel-based treatment in previous studies.

RSID	Gene	Chr	BP	Allele		MCBDR cohort				N08Cx cohort				Cited Articles
				Ref	Alt	Freq.	Beta/Effect	SE	*p*-Value	Freq.	Beta/Effect	SE.	*p*-Value	
rs1056836	*CYP1B1*	2	38298203	C	G	0.596	−1.00	1.15	0.38	0.541	−2.08	1.12	0.06	[19]
rs10771973	*FGD4*	12	32792974	G	A	0.283	−0.15	1.22	0.90	0.313	2.44	1.10	0.03	[21]
rs8187710	*ABCC2*	10	101611294	G	A	0.062	−3.34	2.46	0.17	0.067	−1.79	2.16	0.41	[19]
rs4141404	*LIMK2*	22	31675185	A	C	0.697	−0.21	1.24	0.87	0.734	2.45	1.18	0.04	[26]
rs11572080	*CYP2C8*	10	96827030	C	T	0.112	0.00	1.77	1.00	0.095	−2.81	1.83	0.13	[54,55]
rs17222723	*ABCC2*	10	101595996	T	A	0.062	−3.31	2.46	0.18	0.055	−0.38	2.32	0.87	[19]
rs17781082	*GRIP1/CAND1*	12	67476327	C	T	0.409	0.38	1.31	0.77	0.384	1.12	1.12	0.32	[21]
rs10509681	*CYP2C8*	10	96798749	T	C	0.114	0.23	1.72	0.90	0.095	−2.73	1.83	0.14	[54,55]
rs4737264	*XKR4*	8	56111322	A	C	0.223	2.82	1.47	0.06	0.213	−0.98	1.30	0.45	[21,26]
rs1903216	*BCL6*	3	187629503	A	G	0.510	−0.10	1.25	0.93	0.546	−0.75	1.08	0.49	[21]
rs2233335	*NDRG1*	8	134261065	T	G	0.363	0.19	1.27	0.88	0.341	0.69	1.12	0.54	[21]
rs7001034	*MIR4288 -FZD3*	8	28363378	A	G	0.603	−0.59	1.15	0.61	0.563	−0.04	1.07	0.97	[21]
rs16916932	*CACNB2*	10	18476276	C	T	0.079	−1.57	2.07	0.45	0.063	2.75	2.15	0.20	[21]
rs8110536	*C19orf21*	19	756985	T	G	0.162	−1.66	2.18	0.45	0.150	0.21	1.47	0.89	[26]
rs10932374	*ERBB4*	2	212244403	G	A	0.231	0.18	1.36	0.90	0.248	−0.56	1.20	0.64	[26]
rs17683288	*ARHGEF10*	8	1877480	T	G	0.063	−4.55	3.02	0.13	0.059	1.62	2.15	0.45	[36,37]
rs7833751	*FZD3*	8	28362792	T	G	0.598	−0.81	1.15	0.48	0.539	0.31	1.04	0.77	[21]
rs2032582	*ABCB1*	7	87160618	A	C	0.556	−0.64	1.20	0.59	0.589	0.89	1.09	0.41	[19]
rs7349683	*EPHA5*	4	66197804	C	T	0.345	0.25	1.30	0.85	0.329	−0.53	1.12	0.64	[21,26]
rs9657362	*ARHGEF10*	8	1833801	G	C	0.147	−3.24	1.96	0.10	0.138	1.66	1.53	0.28	[36,37]
rs1045642	*ABCB1*	7	87138645	A	G	0.470	0.20	1.16	0.86	0.502	−0.05	1.05	0.96	[19]

Chr: chromosome; SE: standard error; Freq: frequency. SNP positions on chromosome were based on the GRCh37 (Genome Reference Consortium Human Build 37) assembly in National Center for Biotechnology Information (NCBI), RSID: Reference SNP cluster ID.

**Table 8:** Candidate SNPs associated with CIPN from oxaliplatin-based treatment in previous studies.

RSID	Gene	Chr	BP	Allele		MCBDR Cohort				N08Cx Cohort				Cited Articles
				Ref	Alt	Freq.	Beta/Effect	SE	*p*-Value	Freq.	Beta/Effect	SE.	*p*-Value	
rs34116584	*AGXT*	2	241808314	C	T	0.190	1.65	1.54	0.29	0.179	3.03	1.38	0.03	[61]
rs797519	*DLEU7*	13	51231132	G	C	0.417	−1.45	1.26	0.25	0.392	−1.60	1.10	0.15	[33]
rs4936453	*BTG4*	11	111300782	T	G	0.339	−2.55	1.27	0.04	0.326	−0.50	1.16	0.67	[33]
rs1695	*GSTP1*	11	67352689	A	G	0.374	−1.04	1.17	0.38	0.358	−1.54	1.12	0.17	[62]
rs843748	*ACYP2*	2	54502912	G	A	0.508	0.12	1.15	0.92	0.426	2.04	1.07	0.06	[33]
rs6924717	*FARS2*	6	5304851	C	T	0.156	0.07	1.61	0.97	0.140	2.57	1.52	0.09	[33]
rs2338	*FOXC1*	6	1573613	G	A	0.278	2.33	1.44	0.11	0.280	0.21	1.18	0.86	[33]
rs3212986	*ERCC1*	19	45912736	C	A	0.264	0.67	1.29	0.61	0.258	1.33	1.21	0.27	[63]
rs1138272	*GSTP1*	11	67353579	C	T	0.102	−2.08	1.89	0.27	0.075	−0.53	2.07	0.80	[64]
rs12632942	*SCN10A*	3	38764998	A	G	0.258	0.65	1.37	0.63	0.255	0.85	1.20	0.48	[53]
rs2302237	*SCN4A*	17	62048707	C	T	0.400	−1.12	1.33	0.40	0.363	1.76	1.14	0.12	[53]
rs25487	*XRCC1*	19	44055726	T	C	0.643	−1.89	1.18	0.11	0.659	0.72	1.11	0.52	[65]
rs10486003	*TAC1*	7	97229778	C	T	0.102	0.05	2.35	0.98	0.086	−1.34	1.79	0.45	[33]
rs2230641	*CCNH*	5	86695274	A	G	0.232	−1.48	1.41	0.30	0.200	0.36	1.36	0.79	[22]
rs3114018	*ABCG2*	4	89064581	A	C	0.557	0.85	1.22	0.49	0.487	−0.13	1.09	0.90	[22]
rs17140129	*FARS2*	6	5298362	A	G	0.151	0.72	1.62	0.66	0.128	−0.39	1.61	0.81	[33]

Freq: frequency; Chr: chromosome; SE: standard error; Freq: frequency. SNP positions on chromosome were based on the GRCh37 (Genome Reference Consortium Human Build 37) assembly in National Center for Biotechnology Information (NCBI), RSID: Reference SNP cluster ID.

## Data Availability

The datasets used and/or analyzed during the current study are available from the corresponding author on reasonable request.
